# Far-field polarization signatures of surface optical nonlinearity in noncentrosymmetric semiconductors

**DOI:** 10.1038/s41598-020-67186-0

**Published:** 2020-06-29

**Authors:** A. V. Pakhomov, F. J. F. Löchner, L. Zschiedrich, S. Saravi, M. Hammerschmidt, S. Burger, T. Pertsch, F. Setzpfandt

**Affiliations:** 1 JCMwave GmbH, 14050 Berlin, Germany; 2 Institute of Applied Physics, Abbe Center of Photonics, Friedrich Schiller University Jena, 07745 Jena, Germany; 3 Zuse Institute Berlin, 14195 Berlin, Germany; 4 Fraunhofer Institute for Applied Optics and Precision Engineering, 07745 Jena, Germany

**Keywords:** Nanophotonics and plasmonics, Nonlinear optics

## Abstract

We analyse possibilities to quantitatively evaluate the surface second-order optical nonlinearity in noncentrosymmetric materials based on polarization-resolved analysis of far-field radiation patterns of second-harmonic generation. We analytically demonstrate that for plane-wave illumination the contribution to the second-harmonic signal from the surface of a nonlinear medium exhibits different polarization properties and angular dependencies compared to the contribution from the bulk. In view of this, we optimize the illumination geometry in order to enable the most efficient separation and comparison of both nonlinearities. Furthermore, we consider the illumination of an AlGaAs slab by a tightly-focused linearly-polarized Gaussian beam as an alternative measurement geometry. It is found that the reliable separation of the surface nonlinearity contribution as well as a wide range of detectable values can be achieved with this geometry as well.

## Introduction

Second-harmonic generation (SHG) is the first studied second-order nonlinear effect, where two photons of an incident light beam are nonlinearly coupled to create a photon with twice the frequency of the initial ones^1^. In recent years, SHG in nanoscale structures has gained increasing interest due to their possibility to confine energy in very small volumes, enhancing the overall SHG efficiency^2,3^. Semiconductor materials are advantageous in this regard as they can have very small dissipative losses in specific spectral ranges and feature strong second-order nonlinearities in noncentrosymmetric semiconductors^4^. In contrast to plasmonic devices, where the electric field is concentrated in the vicinity of the metal surface, the relatively low absorption of semiconductors allows the optical field to penetrate deep into the bulk of the material. This fact leads to a much larger fraction of the spatial volume of the nanostructures, where light-matter interaction can take place. Therefore, the use of semiconductors allows for increasing the conversion efficiency of SHG by several orders of magnitude compared to the values achievable in plasmonics^5,6^.

SHG usually originates from two main sources, namely bulk and surface optical nonlinearities. The interplay of these sources is expected to be especially complicated in nanophotonics, since the surface-to-volume ratio is largely increased in the nanostructures. In centrosymmetric materials, like metals, amorphous dielectrics, and many semiconductors (e.g., silicon), the electric-dipole bulk second-order nonlinearity vanishes^7^ and surface contributions become the dominant source of second-order nonlinearity. Due to this fact, SHG has become a powerful technique for probing surfaces and interfaces^8^. However, even in centrosymmetric dielectric materials, the bulk contribution from higher-order multipole terms can become comparable with the surface contribution due to the excitation of optical resonances^9^. Therefore, efforts were undertaken to separate and compare both nonlinear sources. In particular, it was shown that surface and bulk contributions to SHG in isotropic materials can be identified unambiguously by their polarization signatures^10,11^. Based on these theoretical findings, bulk and surface nonlinear responses were experimentally separated and compared using a two-beam technique in poled polymer films^10^ and in thin gold films^11^. The obtained results showed the dominating surface response, although bulk terms can make an appreciable contribution under certain conditions.

In contrast to centrosymmetric materials, for noncentrosymmetric ones the surface SHG is commonly assumed to be negligible with respect to the bulk contribution^12^. However, as some recent studies demonstrate, this is not always the case. Comparable surface and bulk contributions to the SHG signal due to strong interband resonances were observed at semi-insulating and N^+^-doped GaAs-oxide interfaces^13^. In ref. ^14^ the spectrum of the second-order nonlinear susceptibility of GaAs was experimentally measured in a wide frequency range and a strong contribution of surface resonances was observed. A significant surface contribution to SHG was also shown in a number of experiments at the interface between GaAs and aqueous electrolyte^15–17^. In refs. ^18,19^ the SHG from GaP nanopillars was measured and the surface nonlinear response was found to dominate for small enough nanostructures with diameters below $200$ nm. In ref. ^20^ it was found that resonantly enhanced SHG from GaAs dielectric metasurfaces cannot be adequately described when assuming only bulk nonlinearity, thus it was concluded that the surface nonlinearity gives a noticeable contribution. Finally, strong surface-bulk interference was recently observed in the sum-frequency generation from a GaAs crystal with incident visible and infrared pulses^21^. It is also worth to note that the static electric fields within the surface depletion region of a doped GaAs was found to largely enhance the surface-like SHG response from the near-surface region which contributes significantly to the observed SHG and can even dominate in highly-doped samples^22,23^. The above-listed findings point out that at least for pump frequencies close to the surface resonances and/or for nanoscale structures and very thin films with large surface to bulk ratio, the surface nonlinearity cannot generally be neglected and can even have the largest contribution to the total nonlinear response. Therefore, a quantitative evaluation of the surface optical nonlinearity is essential for the design and application of nonlinear optical nanostructures.

The separation and quantitative evaluation of surface and bulk nonlinearities in noncentrosymmetric materials is challenging and only few works have addressed this issue. The idea of polarization-resolved identification of surface nonlinearities in noncentrosymmetric semiconductors based on distinct symmetry properties of the surface and the bulk of a crystal was first proposed by Stehlin *et al*.^24^. They showed theoretically and confirmed experimentally, that for a specific polarization of the exciting wave the total nonlinear response of GaAs can solely be determined by the surface nonlinearity. Following a similar idea, Hollering^25^ and Takebayashi *et al*.^26^ experimentally separated and compared the surface and bulk nonlinear optical responses in GaAs crystals. The rotation-angle dependence of the surface SHG intensity from noncentrosymmetric cubic crystals for different surface-to-bulk ratios was considered in refs. ^27,28^ and the possible influence of the surface orientation on the rotational symmetry was analysed. The surface nonlinearity was experimentally estimated in GaP nanopillars in ref. ^29^ using considerably simplified assumptions regarding the relative contributions of both nonlinearities to the total SHG pattern. However, the varying normal directions of the nanopillar sidewalls significantly complicate the identification and quantification of the surface response.

Despite all these works, detailed studies on the possibility to separate and evaluate bulk and surface SHG in noncentrosymmetric media are still lacking. In particular, even though some authors extracted qualitative estimates of the strength of the surface nonlinearity from experimentally measured rotation-angle dependencies of SHG intensity^25,26^, no attempts have been made to optimize these approaches with respect to their sensitivity. At the same time better understanding of the interplay and mutual effect of surface and bulk nonlinearities in semiconductor nanostructures appears essential for improving the design of efficient nonlinear nanoscale components.

In this work, we make an attempt to fill this gap and consider the possibility to quantitatively compare the bulk and surface contributions to SHG in noncentrosymmetric semiconductors. Our approach is based on different polarization signatures of bulk and surface optical nonlinearities in the far-field optical response of a flat interface. We show theoretically that under plane-wave illumination both contributions to SHG exhibit a distinct dependence on the incidence angles and polarization of the incident wave as well as the angle of crystal axis rotation provided by the specific symmetry properties of the corresponding nonlinear tensors. This allows us to analyse the lower limit of detectable surface contribution to the SHG response and to optimize the illumination parameters to maximize the sensitivity of this approach.

Furthermore, we perform numerical studies of alternative illumination conditions. Specifically, we extend our consideration towards the experimentally relevant case of a planar AlGaAs-air interface illuminated by a tightly-focused linearly-polarized Gaussian beam. We show that the surface and bulk contributions can be unambiguously separated and quantitatively compared from the polarization-resolved far-field radiation patterns of SHG within a wide range of surface-to-bulk ratios, which spans over at least three orders of magnitude. The applicability of our approach could be further boosted by devising special configurations of the illumination field allowing to selectively increase the surface response in the total SHG field.

The paper is organized as follows. In Section 1 we describe the basic idea of our approach and show the results of an analytical treatment of SHG under plane-wave illumination. Section 2 investigates through numerical simulations the case that a tightly-focused linearly-polarized Gaussian beam is used as the illumination source, as well as provides the discussion of the obtained results. Finally, concluding remarks are given in Section 3.

## Model and analytical solution

**Figure 1:**
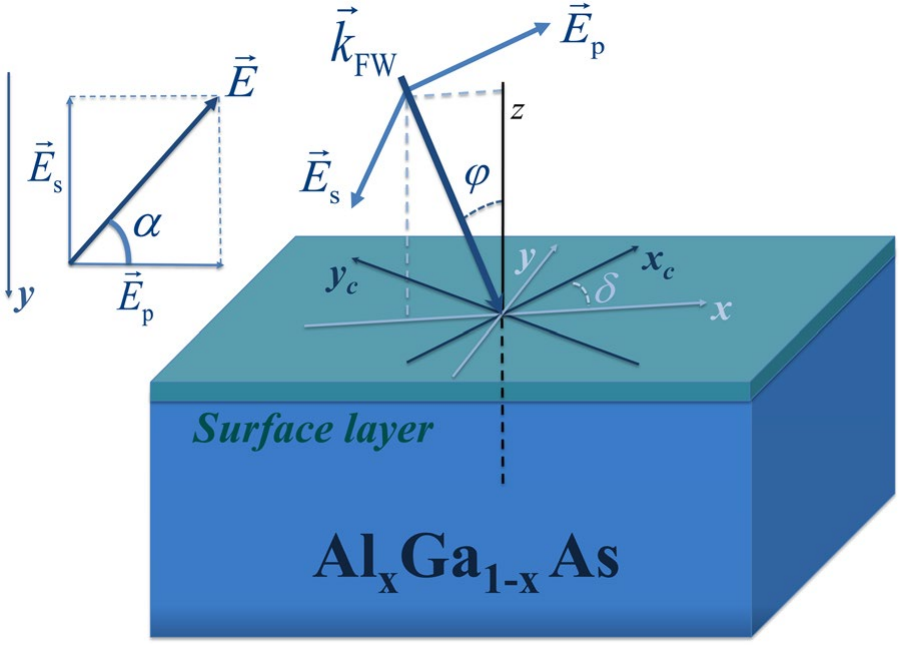
Schematic picture of the considered layout. ${\overrightarrow{k}}_{{\rm{FW}}}$ stands for the wavevector of the fundamental (pump) wave. The inset at the upper left shows the polarization of the fundamental plane wave, where ${\overrightarrow{E}}_{{\rm{s}}}$ and ${\overrightarrow{E}}_{{\rm{p}}}$ correspond to s- and p-polarized field components, respectively.

In this manuscript, we are considering an unstructured interface between air and a semi-infinite nonlinear medium. In the simplest case that we first study, the interface is illuminated by a plane wave. The setup and relevant parameters are schematically depicted in Fig. 1. The illuminating fundamental wave (FW) is characterized by a propagation direction denoted by the wavevector ${\overrightarrow{k}}_{{\rm{FW}}}$, which is incident under an angle $\varphi$ with respect to the surface normal along the *z*-direction. The plane of incidence, defined by the surface normal along the *z*-direction and the propagation constant ${\overrightarrow{k}}_{{\rm{FW}}}$, encompasses the *x*-axis of the beam coordinate system, i.e., the FW is always propagating parallel to the *xz*-plane of this coordinate system. The FW polarization, which is assumed to be linear, can be decomposed in an ${\overrightarrow{E}}_{{\rm{p}}}$ component in and an ${\overrightarrow{E}}_{{\rm{s}}}$ normal to the plane of incidence. The polarization direction is described by the polarization angle $\alpha$ between the electric field vector $\overrightarrow{E}$ and ${\overrightarrow{E}}_{{\rm{p}}}$. The fields in the beam coordinate system are defined by:

1 $$\begin{array}{rcl}{E}_{x}^{(\omega )} & = & {E}_{{\rm{p}}}\,\cos \,\varphi =E\,\cos \,\alpha \,\cos \,\varphi ,\\ {E}_{y}^{(\omega )} & = & {E}_{{\rm{s}}}=E\,\sin \,\alpha ,\\ {E}_{z}^{(\omega )} & = & {E}_{{\rm{p}}}\,\sin \,\varphi =E\,\cos \,\alpha \,\sin \,\varphi .\end{array}$$

To describe a rotation of the plane of incidence with respect to the crystalline lattice of the nonlinear material, we furthermore introduce the coordinate system of the crystal $({x}_{c},{y}_{c},{z}_{c})$. We assume, that the *z*-axis of the nonlinear crystal is oriented normal to the interface, ${z}_{c}=z$, which is a typical case in nanophotonic applications. The in-plane rotation between the beam coordinate system and the crystal coordinate system is described by the angle *δ*. The field components in the crystal coordinate system are given by

2 $$\begin{array}{rcl}{E}_{{x}_{c}}^{(\omega )} & = & {E}_{x}^{(\omega )}\,\cos \,\delta +{E}_{y}^{(\omega )}\,\sin \,\delta ,\\ {E}_{{y}_{c}}^{(\omega )} & = & -{E}_{x}^{(\omega )}\,\sin \,\delta +{E}_{y}^{(\omega )}\,\cos \,\delta ,\\ {E}_{{z}_{c}}^{(\omega )} & = & {E}_{z}^{(\omega )}.\end{array}$$

The generated second-harmonic wave (SHW) is driven by a nonlinear polarization induced by the incident FW beam and the nonlinear susceptibility of the considered structure ${\chi }_{ijk}^{(2)}(\overrightarrow{r})$. Here, the nonlinear susceptibility consists of two parts originating from the bulk and surface contributions, i.e., ${\chi }_{ijk}^{(2)}={\chi }_{ijk}^{{\rm{bulk}}}+{\chi }_{ijk}^{{\rm{surf}}}$. Hence, the nonlinear polarization can be written in the crystal coordinate system as:

3 $$\begin{array}{rcl}{P}_{i}^{{\rm{NL}}}(2\omega ,\overrightarrow{r}) & = & {\varepsilon }_{0}{\chi }_{ijk}^{(2)}(\overrightarrow{r}){E}_{j}^{(\omega )}(\overrightarrow{r}){E}_{k}^{(\omega )}(\overrightarrow{r})\\  & = & {\varepsilon }_{0}{\chi }_{ijk}^{{\rm{bulk}}}(\overrightarrow{r}){E}_{j}^{(\omega )}(\overrightarrow{r}){E}_{k}^{(\omega )}(\overrightarrow{r})\\  &  & +\,{\varepsilon }_{0}{\chi }_{ijk}^{{\rm{surf}}}(\overrightarrow{r}){E}_{j}^{(\omega )}(\overrightarrow{r}){E}_{k}^{(\omega )}(\overrightarrow{r})\\  & = & {P}_{{\rm{bulk}},i}^{{\rm{NL}}}(2\omega ,\overrightarrow{r})+{P}_{{\rm{surf}},i}^{{\rm{NL}}}(2\omega ,\overrightarrow{r}),\end{array}$$

where $i,j,k\in {x}_{c},{y}_{c},{z}_{c}$. The calculated nonlinear polarization is then back-transformed to the beam coordinate system using

4 $$\begin{array}{rcl}{P}_{x}^{{\rm{NL}}} & = & {P}_{{x}_{c}}^{{\rm{NL}}}\,\cos \,\delta -{P}_{{y}_{c}}^{{\rm{NL}}}\,\sin \,\delta ,\\ {P}_{y}^{{\rm{NL}}} & = & {P}_{{x}_{c}}^{{\rm{NL}}}\,\sin \,\delta +{P}_{{y}_{c}}^{{\rm{NL}}}\,\cos \,\delta ,\\ {P}_{z}^{{\rm{NL}}} & = & {P}_{{z}_{c}}^{{\rm{NL}}}.\end{array}$$

For our analysis, we assume that the nonlinear material is a III-V semiconductor, which is a common material class for nonlinear nanooptics. III-V semiconductors feature a zinc-blende crystal structure, which belongs to the $\bar{4}$3*m* symmetry group. Based on this assumption, we can simplify the considered nonlinear tensor.

The bulk nonlinear tensor in III-V semiconductors possesses only nonzero elements if all indices are different, i.e., ${\chi }_{ijk}^{(2)}\ne 0$ if $i\ne j\ne k$. Besides that, such crystal symmetry forces all these nonzero elements to be equal: ${\chi }_{{x}_{c}{y}_{c}{z}_{c}}^{(2)}={\chi }_{{x}_{c}{z}_{c}{y}_{c}}^{(2)}={\chi }_{{y}_{c}{x}_{c}{z}_{c}}^{(2)}$ = ${\chi }_{{y}_{c}{z}_{c}{x}_{c}}^{(2)}$ = ${\chi }_{{z}_{c}{x}_{c}{y}_{c}}^{(2)}={\chi }_{{z}_{c}{y}_{c}{x}_{c}}^{(2)}={\chi }_{{\rm{bulk}}}^{(2)}$. Furthermore, the bulk nonlinearity is homogeneous throughout the nonlinear medium.

On the other hand, the surface nonlinearity is introduced due to the symmetry breaking felt by the few atomic layers directly at the surface. This thin layer exhibiting surface nonlinearity can be regarded either as having very small but finite thickness or as an exactly two-dimensional surface source^30^. Moreover, the surface nonlinear polarization source can be placed on any side of the interface provided that the corresponding rescaling of the nonlinear tensor ${\chi }^{{\rm{surf}}}$ is done^11^. We will assume in the following an exactly two-dimensional source, i.e., we suppose in Eq. (3):

$${\chi }_{ijk}^{{\rm{surf}}}(\overrightarrow{r})={\chi }_{ijk}^{2{\rm{D}}}\cdot \delta (z),$$

and assume that the surface dipole sheet is placed just above the medium interface on the air side. Breaking of the crystal’s bulk symmetry at the interface results in different symmetry properties for the surface layer. Since in our case the *z*-axis is pointing along the surface normal and coincides with one of the crystal axes, we get an *mm*2 symmetry for the surface layer. This symmetry group has the following nonzero components of the nonlinear susceptibility tensor: ${\chi }_{{z}_{c}{z}_{c}{z}_{c}}^{(2)}$, ${\chi }_{{z}_{c}{x}_{c}{x}_{c}}^{(2)}$, ${\chi }_{{z}_{c}{y}_{c}{y}_{c}}^{(2)}$, ${\chi }_{{x}_{c}{z}_{c}{x}_{c}}^{(2)}$, ${\chi }_{{x}_{c}{x}_{c}{z}_{c}}^{(2)}$, ${\chi }_{{y}_{c}{y}_{c}{z}_{c}}^{(2)}$ and ${\chi }_{{y}_{c}{z}_{c}{y}_{c}}^{(2)}$^31^. Following the experimental results of^18–20,29^, the tensor component ${\chi }_{{z}_{c}{z}_{c}{z}_{c}}^{(2)}$ is expected to give the major contribution to the overall surface nonlinear response. This fact also has a simple physical explanation, since only the component of the fundamental field normal to the interface ${E}_{z}^{(\omega )}$ undergoes a jump at the interface and the material properties at the interface have a discontinuity along $z$-axis. For this reason, from this point on we will limit our description of the surface nonlinearity to this term only.

Our aim is to find optimized conditions for the measurement of the SHW generated by the surface nonlinearity. In general, one can assume that the SHW from the surface is almost equally emitted towards the air and the substrate directions. On the other hand, as the exciting FW is propagating into the substrate, the SHW due to the bulk nonlinearity will be predominantly generated with propagation direction into the substrate. Bulk SHG in reflection, i.e., propagating towards air, will be much smaller as the phase mismatch between FW and SHW is very large due to opposite propagation directions. Hence, we will only analyze the reflected SHW in the air domain, as here the influence of the surface nonlinearity will be much larger.

According to Sipe *et al*.^7^, for excitation with a FW plane wave, both nonlinear sources emit only one plane wave component of the SHW in reflection. The *s*- and *p*-polarization components of the amplitude of this SHW propagating upwards are^7^

5 $$\begin{array}{ll}{E}_{{\rm{s}},{\rm{surf}}}^{(2\omega )}=0, & {E}_{{\rm{p}},{\rm{surf}}}^{(2\omega )}=\frac{4i{k}_{{\rm{FW}}}^{2}{\varepsilon }_{2\omega }{P}_{{\rm{surf}},z}^{{\rm{NL}}}\,\sin \,\varphi }{{W}_{0}{\varepsilon }_{2\omega }+{W}_{2}};\\ {E}_{{\rm{s}},{\rm{bulk}}}^{(2\omega )}=-\,\frac{4{k}_{{\rm{FW}}}^{2}{P}_{{\rm{bulk}},y}^{{\rm{NL}}}}{{\varepsilon }_{0}({W}_{0}+{W}_{2})({W}_{2}+2{W}_{1})}, & {E}_{{\rm{p}},{\rm{bulk}}}^{(2\omega )}=-\,\frac{4{k}_{{\rm{FW}}}^{2}({P}_{{\rm{bulk}},x}^{{\rm{NL}}}\sqrt{{\varepsilon }_{2\omega }-{\sin }^{2}\,\varphi }+{P}_{{\rm{bulk}},z}^{{\rm{NL}}}\,\sin \,\varphi )}{\sqrt{{\varepsilon }_{2\omega }}{\varepsilon }_{0}({W}_{0}{\varepsilon }_{2\omega }+{W}_{2})({W}_{2}+2{W}_{1})};\end{array}$$

with

6 $${W}_{0}=2{k}_{{\rm{FW}}}\,\cos \,\varphi ,\,{W}_{1}={k}_{{\rm{FW}}}\sqrt{{\varepsilon }_{\omega }-{\sin }^{2}\,\varphi },\,{W}_{2}=2{k}_{{\rm{FW}}}\sqrt{{\varepsilon }_{2\omega }-{\sin }^{2}\,\varphi }.$$

Using these equations, we can calculate the intensity of the generated SHW. As a specific example material, we assume in our studies Al_*x*_Ga_1−*x*_As. The semiconductor AlGaAs is believed to be one of the most promising material platforms for nanophotonic applications due to its direct band gap, operation without two-photon absorption at 1.55 *μ*m for a molar fraction of Al of $x\ge 0.18$, strong second-order nonlinearity as well as mature nanostructuring technology^32,33^. Therefore, resonant semiconductor metasurfaces from Al_*x*_Ga_1−*x*_As are actively investigated for SHG applications^20,32–38^. In the following, we fix the molar fraction of Al to be $x=0.18$ and use the experimental data for the medium dispersion from ref. ^39^. In particular, we use ${\varepsilon }_{\omega }=11.46$ and ${\varepsilon }_{2\omega }=20.44+3.68i$. This influences only the linear properties of the studied system, hence our results regarding the differentiation of bulk and surface nonlinearities will be valid for different values of the Al content *x* as well as for other materials with similar crystalline lattice, e.g., other III-V semiconductors.

**Figure 2:**
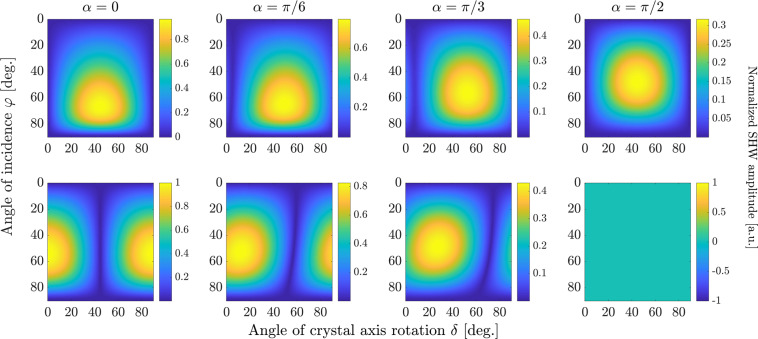
Normalized absolute values of SHW amplitudes generated by bulk nonlinearity, given by Eq. (5), in dependence on the angle of crystal rotation *δ* and the angle of incidence $\varphi$. The upper row shows the *p*-polarized component, the lower row shows the *s*-polarized components. The different columns correspond to different polarization angles *α*. All subplots are normalized to the maximal value among all, which is achieved in the leftmost subplot in the bottom row.

**Figure 3:**
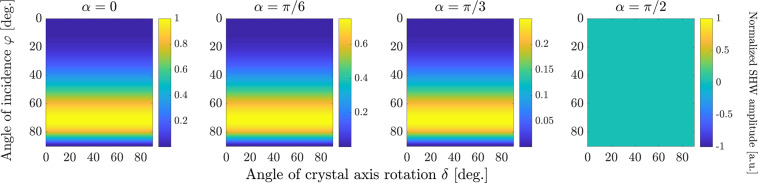
Normalized absolute values of *p*-polarized component of SHW amplitudes generated by surface nonlinearity, given by Eq. (5), in dependence on the angle of crystal rotation *δ* and the angle of incidence $\varphi$. The different columns correspond to different polarization angles *α*. All subplots are normalized to the maximal value among all, which is achieved in the leftmost subplot.

The absolute values of the SHW amplitudes for AlGaAs, calculated with Eq. (5), are plotted in Figs. 2 and 3 for bulk and surface nonlinearity, respectively. The wavelength of the FW was set to $\lambda =1$ *μ*m. Figure 2 shows the amplitude of the backward second-harmonic wave generated by only the bulk nonlinearity. Each column in Fig. 2 is calculated for a specific FW polarization angle *α*, where $\alpha =0$ corresponds to *p*-polarization of the incident plane wave and $\alpha =\pi /2$ corresponds to *s*-polarization. The top and bottom rows correspond to the *p*- and *s*-polarized component of the SHW, respectively. Each panel shows the SHW amplitude in dependence on the incidence angle $\varphi$ of the fundamental plane wave and the angle *δ* between the crystal axis and the plane of incidence (see Fig. 1). We normalize all figures to the maximal value among both field polarizations and all values of *α* in order to illustrate their relative amplitudes. The dependence of the field amplitudes Eq. (5) on the angle *δ* is periodic with a period of 180°. However, the absolute value has a period of only 90°, hence we plot only the range from $\delta =0^\circ$ to $\delta =90^\circ$.

The SHW amplitude in Fig. 2 vanishes in both polarizations for $\varphi =0^\circ$ and $\varphi =90^\circ$. For normal incidence with $\varphi =0^\circ$, the plane wave in the upper domain cannot be excited because such FW excites only components of the electric field parallel to the surface, which due to the properties of the nonlinear tensor in III-V semiconductors can only generate a nonlinear polarization ${P}_{z}^{{\rm{NL}}}$ normal to the surface. Such polarization can only generate fields polarized in *z*-direction, which will not generate a wave propagating towards the air domain. When $\varphi =90^\circ$, the Fresnel reflection coefficient in both polarizations equals 1, i.e., the pump wave is fully reflected and does not induce nonlinear polarization in the bulk. Other points of zero value of the SHW amplitude in Fig. 2 correspond to the values of illumination parameters where one of the components of bulk nonlinear polarization obtained from Eqs. (3 and 4) turns to zero. For example, the SHW in $s$-polarization (bottom row in Fig. 2) becomes zero for those combinations of angles $\varphi$ and *δ*, which give a zero value for the component ${P}_{y}^{{\rm{NL}}}$ in Eq. (4). The fact that for $\alpha  > 0$ the angle *δ* at which the SHW amplitude is zero depends on the incidence angle $\varphi$ is explained by the unequal Fresnel reflection coefficients for both polarization components of the pump plane wave.

Analogous results for only the surface nonlinearity are depicted in Fig. 3. Here, we plot the absolute value of the SHW amplitude only in *p*-polarization since there is no SHW field in *s*-polarization for the employed form of the surface nonlinear susceptibility tensor. Except for the scaling factor due to the varying polarization angle *α*, all plots in Fig. 3 are identical because the surface response is determined only by the *p*-polarized component of the pump wave. They all show no dependence on the crystal rotation angle *δ*, although it is worth noticing that taking account of other smaller components of the surface nonlinear tensor besides ${\chi }_{{\rm{zzz}}}^{2{\rm{D}}}$ would also introduce a dependence of the surface signal on *δ*. The $\varphi$-dependence of the surface-only SHW is due to the joint action of two factors: the Fresnel reflection of the FW and the dependence of the surface nonlinear polarization on the $z$-component of the pump electric field. For $\varphi ={0}^{\circ }$, *E*_*z*_ vanishes, while for $\varphi \to 90^\circ$ the Fresnel transmission coefficient turns to zero, leading to single-peaked dependence with a well-defined maximum of SHW amplitude over $\varphi$.

Our aim is to identify optimal measurement conditions for the determination of the strength of the surface nonlinearity. Assuming that the bulk nonlinearity is known, this can be achieved by solely measuring the ratio of bulk and surface nonlinearities

7 $$\eta =\frac{{\chi }_{{\rm{zzz}}}^{2{\rm{D}}}}{{\chi }_{{\rm{bulk}}}^{(2)}\lambda },$$

with the pump wavelength *λ*. We recall, that all nonzero components of the bulk nonlinear tensor have the same value ${\chi }_{{\rm{bulk}}}^{(2)}$, whereas ${\chi }_{{\rm{zzz}}}^{2{\rm{D}}}$ is the only nonzero component of the surface nonlinear tensor. First insights in suitable measurement conditions can be already obtained by comparison of Figs. 2 and 3. Firstly, we observe that measurements should be done with a FW wave in *p*-polarization, where the effect of the surface nonlinearity does not vanish. Furthermore, the strongest qualitative difference between nonlinear effects induced by bulk and surface nonlinearities appears in their dependence on the crystal rotation angle *δ*, where the surface SHG is constant whereas bulk SHG shows oscillatory behaviour. Hence, we suggest a measurement strategy where the sample is illuminated by a FW plane wave with fixed angles $\varphi$ and *α* and the SH signal is measured in dependence on the crystal rotation *δ*. As seen in Figs. 2 and 3, large values of incidence angles *φ* ~ 50°–70° are needed to achieve stronger SHG signal from both nonlinear sources, while for glancing incidence $\varphi \approx 90^\circ$ or normal incidence $\varphi \approx 0^\circ$ both bulk and surface SHW vanish. Next, we systematically determine optimum values for the angles $\varphi$ and *α* such that the surface contribution to the measured SH dynamics is maximized.

**Figure 4:**
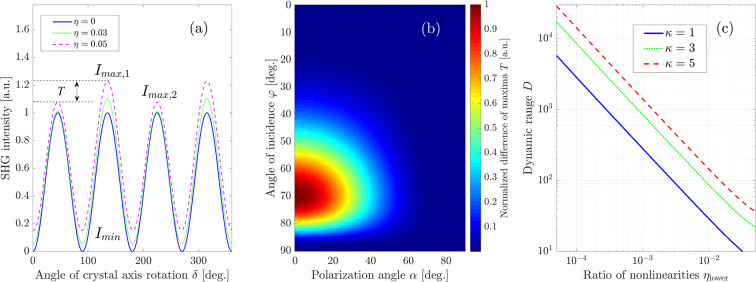
(**a**) Dependence of the SHW intensity *I*_SHG_ on the rotation-angle *δ* for different $\eta$ and parameter values $\varphi =60^\circ$ and $\alpha =0^\circ$, i.e., pure $p$-polarization of the incident plane wave. (**b**) Normalized values of the difference of the maxima shown in (a), defined by Eq. (13), in dependence on the polarization angle *α* and the angle of incidence $\varphi$. (**c**) Minimal dynamic range of a measurement system needed to achieve detection of the surface contribution to the SHW intensity for different ratios $\eta$ of surface and bulk nonlinearity.

We plot the *δ*-dependence of the total SHG intensity for a FW incidence angle $\varphi =60^\circ$ and *p*-polarization with $\alpha =0^\circ$ in Fig. 4(a) for different ratios $\eta$ of bulk and surface nonlinearities. It shows repeating maxima and minima every 90°, however, neighboring maxima show different intensities denoted ${I}_{{\rm{\max }},1}$ for the larger and ${I}_{{\rm{\max }},2}$ for the smaller maximum due to the interference of bulk and surface contributions with a certain phase shift between them. The dependence of the total SHG intensity on the rotation angle *δ* in general has the form

8 $${I}_{{\rm{SHG}}}(\delta ,\alpha ,\varphi )\sim {|{A}_{{\rm{surf}}}(\alpha ,\varphi )+{A}_{{\rm{bulk}}}(\alpha ,\varphi )\sin (2[\delta -{\delta }_{0}])|}^{2},$$

where we separate the dependences on the crystal rotation angle *δ* from the ones on the other angles. To this end, we introduce ${A}_{{\rm{bulk}}}(\alpha ,\varphi )$ and ${A}_{{\rm{surf}}}(\alpha ,\varphi )$ as the complex amplitudes describing the variation of the fields Eq. (5) with the angle *δ*, produced by bulk and surface nonlinear polarizations respectively. ${\delta }_{0}(\varphi ,\alpha )$ corresponds to the shift of the maximum for *A*_bulk_. The dependence of the bulk-only field on 2*δ* directly follows from Eqs. (2–4). Since ${E}_{{\rm{p}},{\rm{surf}}}^{(2\omega )}$ in Eq. (5) does not depend on the angle *δ*, we get

9 $${A}_{{\rm{surf}}}(\alpha ,\varphi )={E}_{{\rm{p}},{\rm{surf}}}^{(2\omega )}(\alpha ,\varphi )=\frac{4i{k}_{{\rm{FW}}}^{2}{\varepsilon }_{2\omega }{P}_{{\rm{surf}},z}^{{\rm{NL}}}(\alpha ,\varphi )\cdot \,\sin \,\varphi }{{W}_{0}{\varepsilon }_{2\omega }+{W}_{2}},$$

while for ${A}_{{\rm{bulk}}}(\alpha ,\varphi )$ and ${\delta }_{0}(\varphi ,\alpha )$ one can obtain the following expressions from Eqs. (2) to (6):

10 $${A}_{{\rm{bulk}}}=-\frac{4{\chi }_{{\rm{bulk}}}^{(2)}{k}_{{\rm{FW}}}^{2}\sqrt{{\Pi }_{1}^{2}+{\Pi }_{2}^{2}}}{{\varepsilon }_{0}({W}_{0}{\varepsilon }_{2\omega }+{W}_{2})({W}_{2}+2{W}_{1})},\,\tan \,(2{\delta }_{0})=-\,\frac{{\Pi }_{1}}{{\Pi }_{2}},$$

with

$$\begin{array}{rcl}{\varPi }_{1} & = & \frac{{\varepsilon }_{0}}{\sqrt{{\varepsilon }_{2\omega }}}({E}_{x}{E}_{y}\,\sin \,\varphi +{E}_{y}{E}_{z}\sqrt{{\varepsilon }_{2\omega }-{\sin }^{2}\,\varphi }),\\ {\varPi }_{2} & = & \frac{{\varepsilon }_{0}}{\sqrt{{\varepsilon }_{2\omega }}}\left(\frac{{E}_{x}^{2}-{E}_{y}^{2}}{2}\,\sin \,\varphi -{E}_{x}{E}_{z}\sqrt{{\varepsilon }_{2\omega }-{\sin }^{2}\,\varphi }\right).\end{array}$$

Once the SHG intensity as a function of *δ* is measured, fitting Eq. (8) to the experimental data would enable to determine the ratio of bulk and surface nonlinearities. The above findings together with the results presented in Figs. 2 and 3 can be considered as the generalization of previous works^25–28^, where only the rotational-angle dependence of SHG response (angle *δ* in our notation) was considered. This fact allows to improve the performance of measurements in terms of the detectable surface contribution by using the dependence on the incidence angle $\varphi$ and polarization angle *α*. Indeed, comparing the dependences of bulk and surface SHW in *p*-polarization on the incidence angle $\varphi$, we can see that for certain combinations of $\varphi$ and the polarization angle *α* one can expect the surface signal to be strongly pronounced against the background of the bulk signal. In particular, in the vicinity of the maximum of the surface contribution, the bulk signal can be much smaller than the pure bulk maximum. Hence, the *δ*-dependence of the total SHG response could be more sensitive to surface contributions and weaker surface nonlinearities can be detectable.

To achieve the highest sensitivity to the contribution of the surface nonlinearity, the difference between the alternating maxima of Eq. (8),

11 $$T=|{I}_{{\rm{\max }},1}-{I}_{{\rm{\max }},2}|,$$

as seen in Fig. 4(a), needs to be maximized. Assuming the ratio between SHG from bulk and surface nonlinearities is small, $|{A}_{{\rm{surf}}}|/|{A}_{{\rm{bulk}}}|\ll 1$, the maxima ${I}_{{\rm{\max }},1}$ and ${I}_{{\rm{\max }},2}$ can be expressed as

12 $$\begin{array}{rcl}{I}_{{\rm{\max }},1} & = & {|{A}_{{\rm{surf}}}(\alpha ,\varphi )|}^{2}+{|{A}_{{\rm{bulk}}}(\alpha ,\varphi )|}^{2}\\  &  & +\,2{|{A}_{{\rm{bulk}}}(\alpha ,\varphi )|}^{2}\cdot |{\rm{Re}}\left(\frac{{A}_{{\rm{surf}}}(\alpha ,\varphi )}{{A}_{{\rm{bulk}}}(\alpha ,\varphi )}\right)|;\\ {I}_{{\rm{\max }},2} & = & {|{A}_{{\rm{surf}}}(\alpha ,\varphi )|}^{2}+{|{A}_{{\rm{bulk}}}(\alpha ,\varphi )|}^{2}\\  &  & -\,2{|{A}_{{\rm{bulk}}}(\alpha ,\varphi )|}^{2}\cdot |{\rm{Re}}\left(\frac{{A}_{{\rm{surf}}}(\alpha ,\varphi )}{{A}_{{\rm{bulk}}}(\alpha ,\varphi )}\right)|,\end{array}$$

where the last terms account for the phase shift between the bulk and surface contributions, which is due to their specific dependence on the complex permittivities. This results in

13 $$T=4{|{A}_{{\rm{bulk}}}(\alpha ,\varphi )|}^{2}\cdot |{\rm{Re}}\left(\frac{{A}_{{\rm{surf}}}(\alpha ,\varphi )}{{A}_{{\rm{bulk}}}(\alpha ,\varphi )}\right)|.$$

In Fig. 4(b) we plot the difference of the SHG maxima *T*, given by Eq. (13) and normalized to its maximum value, versus the polarization angle *α* and the angle of incidence $\varphi$. We can clearly see that this function has a maximum at the optimal parameter values

14 $$\begin{array}{rcl}{\varphi }_{{\rm{optim}}} & \approx  & 70^\circ ,\\ {\alpha }_{{\rm{optim}}} & = & 0^\circ .\end{array}$$

The optimal polarization angle ${\alpha }_{{\rm{optim}}}$ corresponds to *p*-polarized FW excitation as has been discussed before. Importantly, since the function *T* is linearly proportional to ${A}_{{\rm{surf}}}$, the parameters Eq. (14) give the optimum of function Eq. (13) regardless of the value of ${\chi }_{{\rm{surf}}}^{(2)}$, i.e., they correspond to the maximal sensitivity of the described approach in general.

Parameters Eq. (14) provide the optimal measurement conditions to identify the surface signal. Now we aim to quantitatively estimate the detectable values of the surface nonlinearity. Firstly, we notice that in an experimental setup we have to ensure that the difference of alternating maxima in Eq. (13) due to the surface contribution exceeds the noise level in the detection, so that the surface response can reliably be separated from technical noise. This condition can be expressed in mathematical terms through the dynamic range of the detection system *D*, which is the ratio of the saturation signal level to the noise level. Then we can state that to reliably detect the difference of alternating maxima in the *δ*-dependence of the SHW intensity, the requirement

15 $$D > \frac{{I}_{{\rm{\max }},1}}{|{I}_{{\rm{\max }},1}-{I}_{{\rm{\max }},2}|}$$

must be fulfilled. Here we assumed that the maximum value measured in the experiment is given by ${I}_{{\rm{\max }},1}$. If we assume that in a measurement the signal to be measured should be larger than the noise level by a factor $\kappa  > 1$, we arrive at the following condition for the minimum measurable ratio ${\eta }_{{\rm{lower}}}$

16 $$\frac{{I}_{{\rm{\max }},1}}{|{I}_{{\rm{\max }},1}-{I}_{{\rm{\max }},2}|}=\frac{D}{\kappa }.$$

Figure 4(c) shows the lower detectable limit ${\eta }_{{\rm{lower}}}$ versus the dynamic range of the detector calculated by evaluating Eq. (16) for the optimized parameter values Eq. (14) and several exemplary values of $\kappa$. The value of ${\eta }_{{\rm{lower}}}$ for $\kappa =1$ gives the strength of the surface nonlinearity, that our measurement system is in principle capable to measure regardless of the specific experimental scheme. As it could be intuitively expected, larger values of $\kappa$, corresponding to higher accuracy of the experimental results, degrade the sensitivity of the measurements in terms of detectable range of surface nonlinearities. For fixed parameters as given by Eq. (14), the detection threshold ${\eta }_{{\rm{lower}}}$ is almost exactly inversely proportional to the dynamic range *D* and linearly proportional to the accuracy parameter $\kappa$.

The results presented in this section describe an optimized way of measuring surface nonlinearities. However, the optimized plane-wave illumination with large angle of incidence may be hard to achieve experimentally. Thus, we will consider in the following a focused FW beam, which experimentally is readily available and would also yield high conversion efficiency.

## Tightly-focused Gaussian beam illumination

Based on the results of the previous section, we study the same sample geometry with a plane layer of nonlinear material, but illuminated by a normally incident tightly-focused linearly-polarized Gaussian beam (TFGB). This choice is motivated by the above analytical solution of the problem for plane-wave illumination.

First, we have seen in Section 1, that large incidence angles *α* lead to stronger SHG signals from both nonlinear sources. Tight focusing allows to achieve such large incidence angles. Second, we have already noticed that the main difference between surface and bulk signals lies in the dependence of the SHG signal on the angle *δ* between crystal axis and plane of incidence of the illuminating wave. For a normally incident TFGB, the plane-wave Fourier components forming the beam uniformly cover the whole range of values of the angle *δ*. Hence, instead of performing an angular scan of the angle *δ* as for plane-wave illumination, using a TFGB should allow performing the whole quantitative comparison of the nonlinear sources by polarization-resolved analysis of a single measured SHG far-field radiation pattern.

Both bulk and surface SHG excited by tightly focused laser beams have been earlier studied theoretically and experimentally in different media, including isolated centrosymmetric nanospheres^40,41^, metal nano-objects^42^, glass slides^43,44^ and silicon nanowires^45^. The obtained results in particular have shown that analysis of SHG radiation patterns enables separating the second-harmonic response stemming from different elements of the surface nonlinear tensor^40,41,45^, different contributing terms in the bulk quadrupolar response^40,41,43^ or distinguishing the surface and bulk signals^40,44^. However, it is important to emphasize here that all aforementioned works dealt with SHG from centrosymmetric materials only. To the best of our knowledge, SHG under TFGB illumination has not been considered and analysed before for noncentrosymmetric materials.

The case of plane-wave illumination of a single flat medium interface considered in the previous section is the only one which allows for explicit analytical treatment. Finite-sized beams as the now considered TFGBs can in principle also be expanded into multiple plane-wave components, but the nonlinearity in our problem makes the theoretical analysis increasingly cumbersome. Hence, we will now use numerical simulations of the SHG process within the undepleted pump approximation to investigate the interplay of bulk and surface nonlinearity. Nevertheless, the analytic results of the last section provide a framework for the qualitative explanation of the far-field signatures of surface nonlinearity for TFGBs.

**Figure 5:**
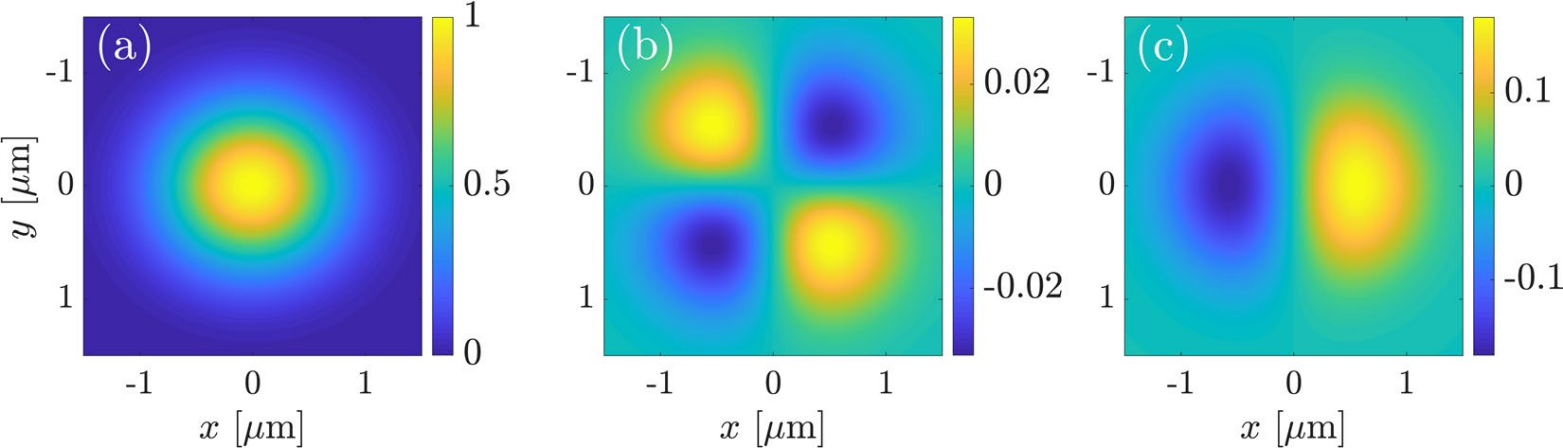
Near-field images of TFGB used in simulations on the transverse plane through the focus: (**a**) *x*-component; (**b**) *y*-component; (**c**) *z*-component of the electric field. Beam waist is equal to ${w}_{0}=750\,{\rm{nm}}$. The beam is linearly polarized along $x$-axis. All field components are normalized to the field value at the beam center.

For our simulations, we use the commercially available finite-element software JCMsuite^46^, assuming a TFGB of the FW as input field. For TFGBs focused to subwavelength waist radius, the paraxial approximation ceases to be sufficient because it does not correctly describe the longitudinal electric field components. Gaussian beam fields which go beyond the paraxial approximation are usually described using expansions into a series of a small parameter $\varepsilon ={w}_{0}/{z}_{R}=2/k{w}_{0} < 1$ (also called Lax series), where ${w}_{0}$ is the beam waist and ${z}_{R}=k{w}_{0}^{2}/2$ is the Rayleigh length^47–52^. In paraxial approximation the defined parameter $\varepsilon$ corresponds to the tangent of the beam divergence angle or, alternatively, the commonly defined numerical aperture of a Gaussian beam. The tighter the beam focusing, the more higher-order correction terms of the Lax series are needed to guarantee the required precision of the calculations. Even though the convergence of the Lax series is in doubt for very tight focusing as $\varepsilon \to 1$, for moderate focusing the truncated Lax series is still expected to yield a reasonable analytic approximation for the fields in TFGB^53^. In our simulations we use a beam waist of ${w}_{0}=750\,{\rm{nm}}$, which corresponds to a parameter value of $\varepsilon \approx 0.42$. The beam waist was placed in the plane of the material interface. According to the relative accuracy estimates in ref. ^49^ and in order to match the accuracy of our finite-element simulations, we add the higher-order corrected terms for linearly-polarized TFGB up to ${\varepsilon }^{3}$ ^51^. Figure 5 illustrates the near-field images of this TFGB used in simulations on the transverse plane through the focus.

**Figure 6:**
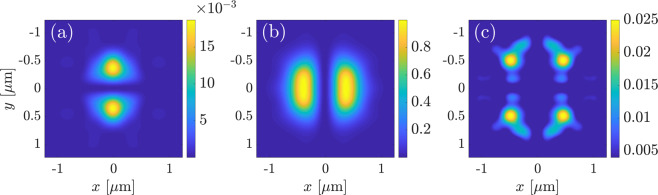
Near-field patterns of the bulk-induced second-harmonic field, calculated in the horizontal plane $z=0.7$ *μ*m above the surface for $\delta =0$: intensities of (**a**) *x*-component; (**b**) *y*-component; (**c**) *z*-component of the electric field. All plots are normalized to the largest value among all, which is achieved in the subplot (**b**).

**Figure 7:**
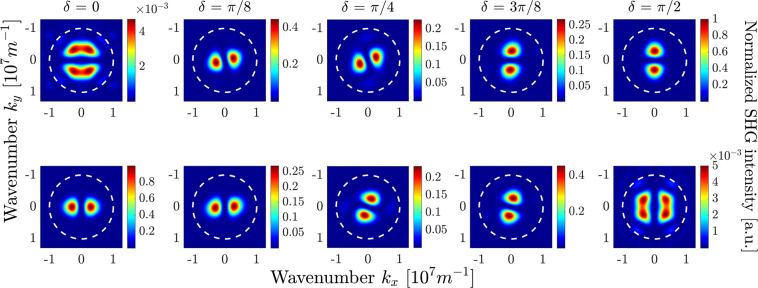
Polarization-resolved bulk-induced far-field SHG intensity distributions for different values of angle $\delta$. Top row: ${x}_{c}$-polarization, bottom row: ${y}_{c}$-polarization. The FW wavelength is $\lambda =1$ *μ*m, thus the SH wavelength is 500 nm. The white dashed circle represents the numerical aperture of a collection lens with $NA=0.8$. All subplots are normalized to the maximal value among all, which is achieved both in the rightmost subplot in the top row and in the leftmost subplot in the bottom row.

The assumed TFGB with normal incidence has only one parameter to scan, the polarization, which is assumed to be linear in the far field. It is described by the angle *δ* with respect to the crystal *x*_*c*_-axis. In Fig. 6 we demonstrate an example of the near-field images of the calculated second-harmonic field for a specific value of the rotation angle $\delta =0$ induced by bulk nonlinearity. Figure 7 shows the corresponding SHG far-field intensity for several polarization angles *δ*. In the upper row, we plot the intensity of the SH polarized along the *x*_*c*_-direction, in the lower row the SH intensity polarized along the *y*_*c*_-direction, taking into account vectorial representation of the SH electric field and the presence of longitudinal field component (see Fig. 6). The plots are normalized to the largest value among all. For illustration purposes, the plots also show a circle corresponding to the numerical aperture of a collecting objective with ${\rm{NA}}=0.8$.

One can see that when the pump beam is polarized along one of the crystal axes, i.e., $\delta =0$ and $\delta =\frac{\pi }{2}$, the amplitudes of the far-field patterns in the two polarizations differ by a factor of around 250. For all values of *δ* we obtain well-pronounced two-lobed field patterns in both polarizations. Such form of the far-field pattern can intuitively be expected due to the specific form of the bulk nonlinear susceptibility tensor. Indeed, e.g., for an *x*_*c*_-polarized TFGB with $\delta =0$, the largest electric field components would be the *x*_*c*_- and *z*_*c*_-fields^51^, resulting in a dominant *y*_*c*_-component of the nonlinear polarization ${\overrightarrow{P}}_{{\rm{bulk}}}^{{\rm{NL}}}$. Such nonlinear polarization results in a dipole emission exhibiting a two-lobed *y*_*c*_-polarized far-field pattern with lobes aligned along the *k*_*x*_ direction, while the *x*_*c*_-polarized field should be much weaker due to the small ${P}_{{\rm{bulk}},{x}_{c}}^{{\rm{NL}}}$. When increasing the angle *δ*, the dominant direction of nonlinear polarization also rotates, whereas the two-lobed shape of the far-field pattern is preserved.

**Figure 8:**
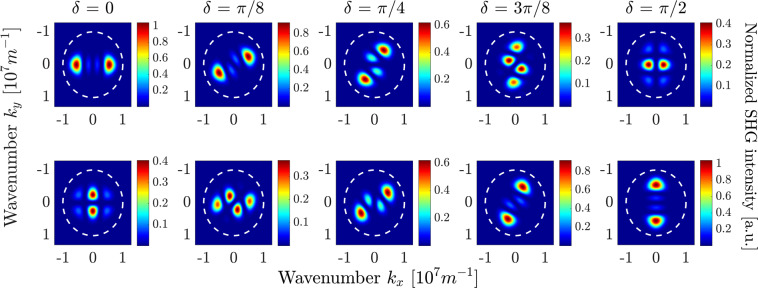
Polarization-resolved surface-induced far-field SHG intensity distributions for different values of angle $\delta$. Top row: ${x}_{c}$-polarization, bottom row: ${y}_{c}$-polarization. The FW wavelength is $\lambda =1$ *μ*m, thus the SH wavelength is 500 nm. The white dashed circle represents the numerical aperture of a collection lens with $NA=0.8$. All subplots are normalized to the maximal value among all, which is achieved both in the leftmost subplot in the top row and in the rightmost subplot in the bottom row.

Far-field intensities for the case of surface-only SHG are depicted in Fig. 8 for *x*_*c*_-(top row) and *y*_*c*_-(bottom row) polarizations of the SH. The plots are scaled again to the maximum value obtained. The far-field patterns here possess different symmetry properties arising from the polarization properties of the illuminating TFGB. Namely, the *z*-component of the TFGB vanishes at the beam symmetry axis and has two maxima located symmetrically along the polarization direction^51^. The dominating ${\chi }_{zzz}^{(2)}$ element of the surface nonlinear susceptibility tensor leads to two domains of nonzero ${P}_{{z}_{c}}^{{\rm{NL}}}$. Emission of both domains interferes, resulting in multi-lobed radiation patterns with comparable far-field amplitudes in both polarizations for all values of the angle *δ*.

As was already mentioned in the previous section, the total SHG far-field pattern can be obtained as the linear superposition of the bulk-only and surface-only fields with a weight factor proportional to the unknown ratio of surface and bulk nonlinear coefficients. We want to analyze the total intensity distribution with regard to a reliable identification of surface-induced and bulk-induced features. Hence, we aim for a configuration that spatially isolates the far-field lobes coming from different sources as much as possible. Comparative analysis of Figs. 7 and 8 shows that these lobes are maximally separated for $\delta =0$ and $\delta =\pi /2$. Specifically, for $\delta =0$, the surface-induced and bulk-induced peaks in the ${x}_{c}$-polarized far-field pattern are symmetrically shifted from the beam axis along the *x*_*c*_- and *y*_*c*_-axis, respectively, thus minimizing their mutual overlap. In the *y*_*c*_-polarized far-field pattern, the overlap of surface-induced and bulk-induced summands is generally larger. However, the contributions are still distinguishable, since the bulk-induced far-field has its main lobes on the *k*_*x*_-axis and vanishes on the *k*_*y*_-axis while just the opposite takes place for the surface-induced far-field.

**Figure 9:**
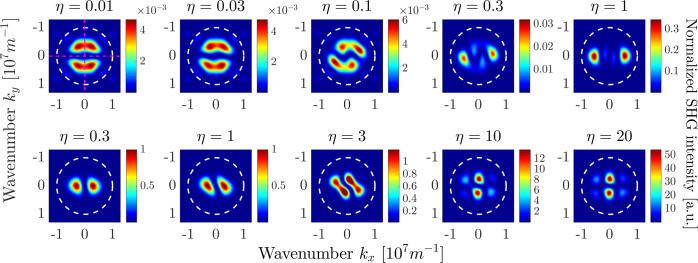
Polarization-resolved far-field SHG intensity distributions for different values of parameter $\eta$ with $\delta =0$. Top row: *x*_*c*_-polarization, bottom row: *y*_*c*_-polarization. The FW wavelength is $\lambda =1$ *μ*m, thus the SH wavelength is 500 nm. The white dashed circle represents the numerical aperture of a collection lens with $NA=0.8$. The dashed magenta lines in the upper left image denote cross sections of the SH intensity, which are plotted in Fig. 10.

**Figure 10:**
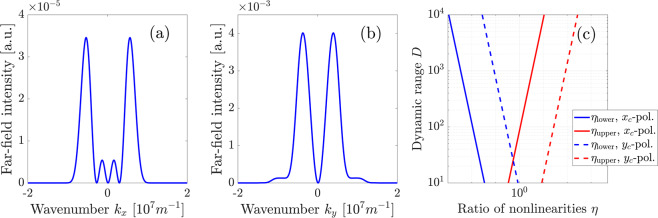
Cross-sections through the far-field intensity pattern in *x*_*c*_-polarization for $\eta =0.01$, marked by the dashed magenta lines in Fig. 9. (**a**) SH intensity along the *k*_*x*_-axis for ${k}_{y}=0$ and (**b**) along the *k*_*y*_-axis for ${k}_{x}=0$. (**c**) The diagram, showing the upper and lower detection thresholds of $\eta$ Eq. (17) vs. the dynamic range of the camera for $\kappa =1$ for *x*_*c*_-polarization and *y*_*c*_-polarization.

Based on this comparative analysis, in the following we investigate the total SH far-field intensity for $\delta =0$, which is depicted in Fig. 9 for different ratios $\eta$ of surface and bulk nonlinear coefficients according to Eq. 7. Again, we plot in the upper row the *x*_*c*_-polarized SH, whereas the lower row shows the *y*_*c*_-polarized SH. All plots are scaled to the largest amplitude in the bulk-induced far-field pattern. Taking into account the largely different amplitudes of the bulk-induced far-field patterns in the two polarizations, different values of $\eta$ are also taken for both polarizations to illustrate the transition from pure-bulk to pure-surface SHG patterns in *x*_*c*_- and *y*_*c*_-polarization, respectively. The observed far-field patterns clearly demonstrate that in each polarization for small values of $\eta$ below a certain threshold the observed far-field coincides with the pure bulk pattern. Increase of $\eta$ results in a gradual transition towards a pure surface field pattern, where the specific field distributions stem from interference of both nonlinear sources. This intermediate range of $\eta$ values is of main interest for us. Each specific far-field intensity distribution with certain relative amplitudes of bulk-induced and surface-induced peaks is unambiguously linked to a specific value of $\eta$. Specifically, for measurement of the surface-to-bulk ratio in *x*_*c*_-polarization, we can use that the maxima induced by surface nonlinearity lie on the axis defined by ${k}_{y}=0$, where the bulk induced SH is zero. In contrast to this, the bulk induced SH is maximal on the axis ${k}_{x}=0$, where no surface SH is generated. Hence, the ratio of maxima along *k*_*x*_- and *k*_*y*_-axis is proportional to $\eta$. Cross-sections of the far-field intensity pattern in *x*_*c*_-polarization for $\eta =0.01$, denoted by the magenta lines in the corresponding plot in Fig. 9, are shown in Fig. 10. With increasing the value of $\eta$, the field distribution in Fig. 10(a), which is solely caused by the surface nonlinear polarization, will grow linearly in amplitude without changing its shape, while the bulk-induced field in Fig. 10(b) does not change. Similar to the previous section, we now want to establish boundaries on the measurable ratios $\eta$. They are given by the ratio of the maximum SH intensities stemming from bulk and surface nonlinearities. Assuming that we consider only signals along the ${k}_{x}=0$ and ${k}_{y}=0$ axes, we obtain two maximum intensities for bulk and surface nonlinearity, respectively. We use ${I}_{1}^{{\rm{\max }}}$ to describe the larger and ${I}_{2}^{{\rm{\max }}}$ to describe the smaller of these values. The detectable range of surface nonlinearities can now be established using the criterion

17 $$\frac{{I}_{1}^{{\rm{\max }}}}{{I}_{2}^{{\rm{\max }}}}=\frac{D}{\kappa },$$

where *D* again is the dynamic range of the measurement system and $\kappa$ is a constant. Equation (17) can be evaluated independently for the far-field SH intensity pattern measured in the *x*_*c*_- and *y*_*c*_-polarizations.

Figure 10(c) depicts the values for $\eta$ where condition Eq. (17) is fulfilled with $\kappa =1$ for both polarizations. These solutions have been calculated numerically using the far-field intensities simulated for bulk and surface nonlinearities. For each polarization, there exists a lower limit ${\eta }_{{\rm{lower}}}$, where ${I}_{2}^{{\rm{\max }}}$ stems from the surface nonlinearity, and an upper limit ${\eta }_{{\rm{upper}}}$ where ${I}_{2}^{{\rm{\max }}}$ is generated by bulk nonlinearity. For example, for dynamic range $D=100$ we find that the lower detectable limit ${\eta }_{{\rm{lower}}}$ in *x*_*c*_-polarization is

$${\eta }_{{\rm{lower}}}\approx 0.01,$$

and the whole the range where the surface nonlinearity can be quantified from the *x*_*c*_-polarized far-field distribution is

$$0.01\lesssim \eta \lesssim 1.$$

In a similar way, from the *y*_*c*_-polarized far-field distribution we get the range

$$0.1\lesssim \eta \lesssim 10.$$

Notably, the range of $\eta$ where the surface nonlinearity can be identified are different for the two polarizations for this particular dynamic range and just slightly overlap. This fact means, that depending on the actual value of $\eta$ in the specific semiconductor material, the far-field signatures of the surface nonlinearity might be predominantly manifested in one polarization only. In total, for $D=100$ we can hence estimate the detectable range of surface-to-bulk ratios from polarization-resolved measurements to span over the values

18 $$0.01\lesssim \eta \lesssim 10,$$

i.e., to cover more than 3 orders of magnitude of $\eta$ values.

The specific values of upper and lower boundaries of the accessible $\eta$ range depend on the refractive indices of the substrate both at the fundamental and the second-harmonic wavelengths, i.e., on the specific material. However, comparing the results for plane-wave and TFGB illumination, we find that the former approach is more sensitive, i.e., the minimum values of $\eta$ where a surface nonlinearity could be detected are about three times larger for the TFGB case. This is due to the specific spatial distribution of the SH generated by the surface nonlinearity, which is not concentrated in a single maximum. The main advantage of the TFGB illumination thus appears to lie in its relative simplicity, since only a single measurement of the far-field SHG radiation pattern in different polarizations is needed without any further movements in the setup.

It would be also interesting to compare the obtained range Eq. (18) with values obtained in the literature. Values of the bulk nonlinear coefficients ${\chi }_{{\rm{bulk}}}^{(2)}$ in noncentrosymmetric semiconductor materials are usually of the order of 10^−12^ m/V, though for III-V semiconductors it achieves ~10^−10^ m/V^14^. The strength of the surface nonlinearity ${\chi }_{{\rm{zzz}}}^{2{\rm{D}}}$ in different solids varies quite strongly^9,11,26,29,54,55^, mainly attaining values in the range ~10^−20^–10^−17^ m^2^/V. For the parameter $\eta$ from Eq. (7) this would give the range $0.01\lesssim \eta \lesssim 10$ for ${\chi }_{{\rm{bulk}}}^{(2)}\sim {10}^{-12}$ m/V and respectively smaller values for larger ${\chi }_{{\rm{bulk}}}^{(2)}$. Along with that, experimental measurements in^29^ for III-V semiconductor, namely GaP nanopillars, yielded in our notation $\eta \sim 0.1$, what allows to expect $\eta$ in other III-V semiconductor to also fall within the same limits. One can see that this estimated range reasonably correlates with the detectable range in Eq. (18).

The most natural approach to extend the detectable range (18) is to devise more complex illuminating sources, mainly to generate larger longitudinal components of the electric field. Stronger longitudinal fields are expected to enlarge the surface contribution to the total far-field pattern since the surface nonlinear polarization scales quadratically with the *E*_*z*_ component of the illuminating beam due to the dominating ${\chi }_{zzz}^{(2)}$ tensor element, in contrast to the bulk nonlinear polarization which scales linearly.

This can be in principle realized by oblique incidence of a TFGB, which seems however quite challenging in the experimental implementation with regard to collecting SHG radiation, as compared to the case of normal incidence. Other options include more complex polarization beam structures, like radially-polarized tightly-focused beams^56–58^, or specially-tailored optical beams possessing large longitudinal components of the electric field, e.g., higher-order cylindrical beams^59^, spatially phase-shaped beams^60^, polarization vortices^61^, photonic nanojets^62^ or optical needles^63,64^. We hope that applying such alternative sources of pump field can allow to sufficiently boost the applicability limits of the proposed method for the quantification of surface nonlinearity.

## Conclusion

Even though the nonlinear optical response of noncentrosymmetric semiconductor materials can be often properly described assuming only the bulk second-order nonlinearity, the contribution of the surface nonlinearity may also play a significant role. Particularly, this happens in certain wavelength ranges, when the absorption of the material at the fundamental frequency is high or in the vicinity of surface resonances, as well as in nanoscale structures with largely increased surface to volume ratio or ultrathin films.

We have shown here that due to different symmetry properties of the bulk crystal and the interface, the surface optical nonlinearity should exhibit specific signatures in the polarization-resolved far-field pattern allowing to separate it from the bulk nonlinear response. Distinct features for plane-wave illumination were represented through the dependence of the SHW amplitude on the incidence angle and polarization of the incident FW plane wave. The obtained results offer the opportunity to distinguish and compare bulk and surface contributions to SHG based on polarization-resolved angular scans of the emitted SHG.

We extended our findings to more realistic illumination conditions and examined SHG from a plane interface under illumination by a tightly-focused linearly-polarized Gaussian beam. It was demonstrated that polarization-resolved analysis of the SHG radiation pattern allows to reliably identify, separate and compare both nonlinear sources. The proposed setup with tightly-focused Gaussian beam illumination enables quantifying surface optical nonlinearities in a wide range of its strengths from just a few polarization-resolved measurements of the far-field SHG radiation pattern. This fact seems to be essential to simplify the experimental measurements.

Despite that all the calculations were performed for an Al_*x*_Ga_1−*x*_As slab, our findings are directly applicable to other widely used III-V semiconductor materials that belong to the $\bar{4}$3*m* crystal symmetry group, e.g., GaAs, GaP, InSb, InAs, AlAs, or InP. The extension to other noncentrosymmetric media is also straightforward and can be done with due regard for the specific type of bulk crystal symmetry. We believe that our results contribute to a better understanding of the nonlinear optical properties of noncentrosymmetric materials at the nanoscale in order to boost the SHG conversion efficiency in semiconductor nanostructures.
